# AI-Supported, Integrative Prediction of Postoperative Delirium: Protocol for the CONFUSED Study

**DOI:** 10.2196/87020

**Published:** 2026-05-25

**Authors:** Katharina Rump, Hartmuth Nowak, Martin Eisenacher, Samuel Busch, Britta Westhus, Matthias Unterberg, Sara Sordon, Lars Palmowski, Andrea Witowski, Malte Bayer, Thilo Bracht, Dominik Ziehe, Michael Adamzik, Tim Rahmel, Björn Koos, Lars Bergmann, Barbara Sitek

**Affiliations:** 1 Ruhr University Bochum, Knappschaft Kliniken Universitätsklinikum Bochum, Klinik für Anästhesiologie, Intensivmedizin und Schmerztherapie, Universitätstraße 150, Bochum, Germany, 49 02343229242; 2 Ruhr University Bochum, Knappschaft Kliniken Universitätsklinikum Bochum, Klinik für Anästhesiologie, Intensivmedizin und Schmerztherapie, Zentrum für Künstliche Intelligenz, Medizininformatik und Datenwissenschaften, Bochum, Germany; 3 Ruhr University Bochum, Medizinisches Proteom-Center, Medizinische Fakultät, Bochum, Germany; 4 Ruhr University Bochum, Knappschaft Kliniken Universitätsklinikum Bochum, Klinik für Anästhesiologie, Intensivmedizin und Schmerztherapie, Clinical Proteomics, Bochum, Germany

**Keywords:** postoperative delirium, POD, delirium, surgery, biomarkers, prediction, artificial intelligence

## Abstract

**Background:**

Postoperative delirium (POD) is a frequent and serious complication in older surgical patients, characterized by acute cognitive dysfunction and fluctuating levels of consciousness. POD is associated with prolonged hospitalization, long-term cognitive decline, reduced quality of life, and increased mortality. Despite its clinical relevance, the underlying pathophysiological mechanisms remain poorly understood, and reliable biomarkers for early prediction and prevention are lacking.

**Objective:**

The CONFUSED study aims to identify molecular and clinical predictors of POD by integrating clinical data with proteomic, transcriptomic, and epigenetic analyses. The primary objective is to develop predictive models for POD using multimodal data. Secondary objectives include the identification of delirium-associated genes, proteins, and epigenetic signatures, as well as the exploration of patient subgroups at increased risk for POD.

**Methods:**

CONFUSED is a prospective observational cohort study conducted at a German university hospital. Adult patients undergoing major surgery under general anesthesia will be enrolled until 100 cases of POD have been observed, which is expected to require a total sample size of approximately 200 to 300 patients. Blood samples are collected at 4 predefined time points: before premedication, immediately after surgery, and on postoperative days 2 and 5. Samples undergo comprehensive proteomic profiling, transcriptomic analysis using RNA microarrays, DNA methylation analysis, and genotyping of selected polymorphisms. Clinical data, including demographics, comorbidities, perioperative variables, medications, and delirium assessments using the Confusion Assessment Method (CAM) and CAM for the intensive care unit, are systematically recorded. Statistical analyses include univariate and multivariate methods, as well as machine learning approaches such as random forests and support vector machines, to identify relevant biomarkers and develop predictive models. The study protocol follows STROBE (Strengthening the Reporting of Observational Studies in Epidemiology) and TRIPOD (Transparent Reporting of a Multivariable Prediction Model for Individual Prognosis or Diagnosis) guidelines and was approved by the responsible ethics committees.

**Results:**

The study was registered in the German Clinical Trials Register (DRKS00033854) on March 18, 2024. Recruitment started in January 2024 and is ongoing at the time of manuscript submission. As of now, 135 patients have been enrolled. Sample collection and laboratory analyses are ongoing. Data analysis began in January 2026, with first results anticipated in July 2026. Final data lock is anticipated after the completion of recruitment.

**Conclusions:**

By integrating multimodal molecular data with clinical parameters and applying advanced machine learning techniques, the CONFUSED study aims to improve the prediction and understanding of POD. The results are expected to support the development of personalized preventive strategies and contribute to improved perioperative care for patients at risk of POD.

## Introduction

Delirium is a common postoperative complication, particularly in patients above the age of 65 years, and its causes are still largely unexplored [1]. Nevertheless, the effects of delirium are very serious for patients and, in some cases, can even drastically affect quality of life and even life expectancy. The incidence of postoperative delirium (POD) in older patients is 5% to 10% on average but can be higher in high-risk procedures such as total joint arthroplasty [2]. POD is therefore a frequent and life-threatening complication. At the center of this clinical picture is a functional disorder of the brain. Patients are affected symptomatically by disorientation, restlessness, hallucinations, or anxiety [3]. Despite these impressive and often serious symptoms, POD is often recognized late, and successful intervention is delayed [4]. However, it should be noted that the more time elapses before the diagnosis is made, the higher the probability of a severe and complicated course of POD. POD is associated with prolonged hospital stays, extended ventilation times, increased care requirements, and higher mortality rates [5]. Recovery from POD has considerable intraindividual variability, ranging from hours to months, and can present as a hypoactive, hyperactive, or mixed subtype, characterized by symptoms such as apathy, confusion, agitation, delusions, and signs of sympathetic arousal [6]. The pathophysiology of POD is not yet fully understood, but it is hypothesized to be related to acute disruptions in neurotransmitter levels and neuroinflammation [1]. Risk factors for POD include preoperative impaired cognitive and general health status, frailty, medical comorbidities such as hypotension and hypoxia, and exposure to psychoactive drugs [7]. Postoperative neuroinflammation, which is a major contributor to the development and progression of POD, can be exacerbated by complications such as infections, pulmonary issues, and hypoxemia [8]. The use of certain anesthetics has also been linked to long-term alterations in brain morphology and function, particularly at the extremes of age [9,10]. It is known from previous studies that the plasma levels of neuron-specific enolase and S-100 β protein may correlate with POD [11]. However, there are currently no valid laboratory tests for diagnosis. Furthermore, apart from an avoidance strategy for possible triggering drugs, there are only a few effective preventive and therapeutic approaches [3,12,13]. Existing models rely on clinical variables or single biomarkers. Our study integrates multiomics with advanced machine learning (ML) to explore novel predictive signatures and underlying biological mechanisms.

The aim of this study is to develop a prediction tool for POD in patients undergoing surgery. The study will address the following main objectives:

1. Identification of significant differentially expressed genes and proteins in blood samples through proteomic analyses and RNA microarrays as the basis for the development of predictive models
2. Collection and integration of clinical data
3. Development of a delirium prediction model based on ML, combining clinical data with data from proteomics and genetics

Following these preliminary tasks, the developed predictive models will be validated in a new independent patient cohort. These efforts will extend beyond the planned initial funding. It is expected that the results of the overall study will contribute to the development of new diagnostic tests and clinical decision tools.

## Methods

This study is a prospective observational study conducted in the Department of Anesthesia of a German university hospital. The protocol follows the STROBE (Strengthening the Reporting of Observational Studies in Epidemiology) and TRIPOD (Transparent Reporting of a Multivariable Prediction Model for Individual Prognosis or Diagnosis) guidelines.

### Ethical Considerations

The study was reviewed and approved by the Ethics Committee of the Medical Faculty of Ruhr University Bochum (approval number 23‐7794) and the Ethics Committee of Westfalen-Lippe (approval number 2024‐082-f-S). All participants will be recruited from the anesthesia outpatient clinic and provided written informed consent prior to participation. The study will be conducted in accordance with the Declaration of Helsinki and relevant local regulations. Participant privacy and data confidentiality will be strictly maintained throughout all stages of data collection, analysis, and reporting. No financial compensation will be provided to participants.

### Patient Recruitment

Patients are recruited after registration in the anesthesia outpatient clinic of the Department of Anesthesiology, Intensive Care Medicine, and Pain Therapy. All patients presenting to the preanesthesia clinic are screened for eligibility to participate in the study. Eligible patients undergo medium to major surgical procedures under general anesthesia. This approach ensures that recruitment is as efficient as possible, allowing us to reach a sufficient number of patients with delirium in a timely manner. Patients are recruited based on the inclusion criteria and the absence of exclusion criteria in the preoperative assessment clinic. The study will be concluded once 100 patients with delirium have been enrolled. Patients without delirium form the control cohort. The inclusion and exclusion criteria are provided in Textbox 1.

Textbox 1. **Inclusion and exclusion criteria of the study.**

Inclusion criteria:

- Age ≥75 years
- Delirium Risk Assessment Score ≥5
- Medium to major surgical procedures under general anesthesia, including:
  - Endoprosthetic procedures (hip and knee joint)
  - Traumatological procedures for femoral neck fractures
  - Spinal disc surgery
  - Visceral surgery including cholecystectomy

Exclusion criteria:

- Age <75 years
- Delirium Risk Assessment Score <5
- Existing anticholinergic medication
- Intracerebral intervention
- Preexisting severe dementia
- Pharmacological immunosuppression
- Chronic skin diseases
- Patient refusal
- Previous participation in this study

### Sample Size Considerations

The study is designed as an exploratory, hypothesis-generating investigation with a focus on identifying biomarkers and predictive signatures associated with POD. Rather than relying solely on a fixed total sample size, the study aims to include a sufficient number of patients with incident POD to enable meaningful exploratory analyses.

Specifically, the target is to observe 100 patients who develop POD. Based on historical incidence rates of POD in older surgical patients, including data from a systematic review of 3533 patients across 19 cohort studies [14], the pooled incidence of POD is estimated at approximately 24% (72/300) overall, with variation depending on the type of surgery (mixed noncardiac 23%, orthopedic 27%, and tumor 19%). Given this expected incidence, it is anticipated that approximately 200 to 300 patients will need to be enrolled to achieve the target number of POD cases. Recruitment will therefore continue until the required number of POD cases is reached.

We acknowledge that even with this sample size, high-dimensional omics analyses remain at risk of overfitting. To address this, feature selection, regularization techniques, and nested cross-validation will be applied to enhance the robustness and reproducibility of the findings. As such, the study is explicitly designed as an initial exploratory investigation, and any identified biomarker signatures or predictive models will require validation in larger, independent cohorts.

### Patient Information and Consent

Participation in the study is voluntary. Prior to the start of the study, patients are informed both verbally and in writing about the nature and scope of the planned investigation, with particular emphasis on the potential health benefits and possible risks. Informed consent or assent will be obtained by the principal investigator or a qualified, trained study physician through a face-to-face discussion with the participant or authorized proxy, using the approved information sheet and consent form, ensuring adequate time for questions and written documentation of consent. Consent is documented by the patient’s signature on the consent form. This consent can be withdrawn at any time without giving reasons and without negative consequences for the patient’s further medical care. It should be noted that the patients will not have any direct benefit from the study, but the knowledge gained will benefit future patients. Participants who experience harm related to trial participation will receive appropriate medical care and compensation in accordance with applicable regulations, and provisions for ancillary and posttrial care will be ensured as clinically indicated.

### Methodology

**Figure 1.**
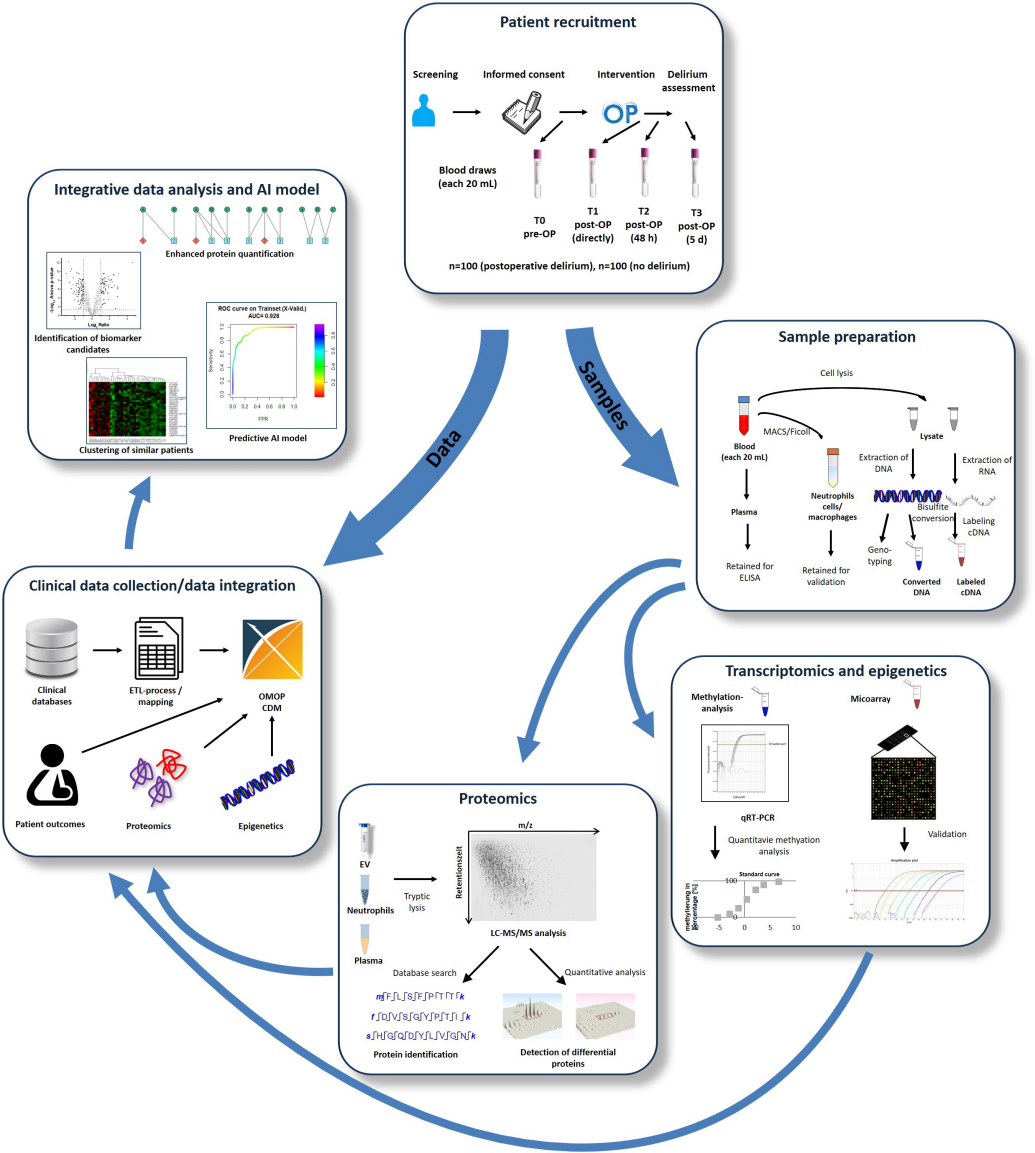
Schematic representation of the planned workflow. Blood samples from patients will be collected at 4 time points. The collected samples will be processed and prepared for further analyses in transcriptomics, epigenetics, and proteomics. Clinical data will be incorporated into the data integration pipeline and processed, so that they can be combined with the results from transcriptomics, epigenetics, and proteomics for the final evaluation in the context of integrative data analysis. AI: artificial intelligence; AUC: area under the curve; cDNA: complementary DNA; ELISA: enzyme-linked immunosorbent assay; ETL: extract, transform, load; EV: extracellular vesicle; FPR: false positive rate; LC-MS/MS: liquid chromatography–tandem mass spectrometry; MACS: magnetic-activated cell sorting; OMOP CDM: Observational Medical Outcomes Partnership Common Data Model; post-OP: postoperative; pre-OP: preoperative; qRT-PCR: quantitative reverse transcription polymerase chain reaction; ROC: receiver operating characteristic.

After obtaining consent, patients are enrolled in the study. The study workflow for each patient is outlined in Table 1 and illustrated in Figure 1.

**Table 1.** Study timeline detailing the timing of various procedures and data collection points throughout the study for each individual patient.

Time point	Study enrollment	Baseline	Preoperative (induction)	Intraoperative	Postoperative (recovery room)	Day 1	Day 2	Day 3	Day 4	Day 5	Hospital discharge	30-day follow-up	90-day follow-up
Study enrollment													
Screening for inclusion and exclusion criteria	✓												
Informed consent	✓												
Measurements													
Blood collection			✓		✓		✓		✓				
Microarray analysis of whole blood RNA		✓			✓								
Epigenetic analysis of whole blood DNA		✓			✓								
Genotyping of whole blood DNA		✓											
Serum/plasma storage		✓	✓	✓	✓		✓		✓				
ELISA^a^ from serum samples		✓	✓	✓	✓		✓		✓				
Isolation of neutrophils*,* PBMCs^b^, and macrophages		✓	✓	✓	✓		✓		✓				
Proteomic analyses		✓	✓	✓	✓		✓		✓				
AChE^c^ measurement		✓	✓	✓	✓	✓	✓	✓	✓	✓			
Clinical data collection													
Demographic and medical baseline data		✓											
Surgery-specific data				✓									
Complete digital anesthesia documentation			✓	✓	✓								
Intraoperative EEG^d^ monitoring				✓									
All clinical parameters		✓	✓	✓	✓	✓	✓	✓	✓	✓	✓		
Complete progress documentation		✓	✓	✓	✓	✓	✓	✓	✓	✓	✓		
Delirium screening (CAM^e^ or CAM-ICU^f^)			✓	✓	✓	✓	✓	✓	✓	✓	✓		
Neuropsychological status (RBANS^g^)											✓	✓	✓
Health-related quality of life (SF-36^h^)											✓	✓	✓
Mortality											✓	✓	✓

a ELISA: enzyme-linked immunosorbent assay.

b PBMSs: peripheral blood mononuclear cells.

c AChE: acetylcholinesterase.

d EEG: electroencephalogram.

e CAM: Confusion Assessment Method.

f CAM-ICU: Confusion Assessment Method for the intensive care unit.

g RBANS: Repeatable Battery for the Assessment of Neuropsychological Status.

h SF-36: 36-item Short-Form Health Survey.

At study inclusion, each patient’s cognitive status is assessed to ensure eligibility and to document baseline cognitive function. In order to determine whether cognitive disorders are already present, a Mini-Mental State Examination and a Delirium Risk Assessment Score (DRAS) test are included as part of the consent process. The DRAS is determined preoperatively in the preanesthesia clinic by evaluating patient-related factors such as age, comorbidities, type of surgery, and medication use. The individual items are summed up to generate a total score, with higher values indicating an increased risk of POD.

Patients will undergo general anesthesia according to the clinic’s standard protocol, which includes the administration of propofol, sufentanil, and, if required, rocuronium for muscle relaxation, as well as maintenance with sevoflurane. Anesthesia depth is strictly monitored using Narcotrend (Narcotrend-Gruppe) with a target anesthesia depth of stages D0-E2. Delirium is assessed preoperatively, upon transfer from the recovery room, and on the 5 postoperative days using the Confusion Assessment Method (CAM), the CAM for the ICU (CAM-ICU), and the Delirium Observation Scale [15,16].

### Blood Sampling and Analysis

In addition, 20 mL of venous whole blood will be collected in ethylenediaminetetraacetic acid, serum, RNA exact, and DNA exact tubes (Sarstedt) at 4 points in time: during premedication, immediately postoperatively, on the second postoperative day, and on the fifth postoperative day. Serum and plasma samples, proteins, DNA, and RNA will be extracted from these whole blood samples. Furthermore, specific cell fractions such as neutrophils, macrophages, and peripheral blood mononuclear cells will be isolated. Proteins will be isolated from these cell samples and, together with plasma, subjected to the proteomic analysis using mass spectrometry to identify potentially significant proteins. Concurrently, an analysis of candidate genes, such as ACHE, BCHE, CHRNA7, chemokines, and cytokines, will be conducted to investigate methylation processes associated with the occurrence of POD. For methylation analysis, DNA samples are subjected to bisulfite conversion, and methylation will be quantified using quantitative polymerase chain reaction with specific primers [17]. The activity of acetylcholinesterase and butyrylcholinesterase will be assessed using ELISA (enzyme-linked immunosorbent assay).

In addition, an in silico analysis of the identified target genes will be performed to identify potential polymorphisms. Polymorphisms that appear potentially relevant will be genotyped in the entire patient cohort using the TaqMan Assay (TaqMan SNP Genotyping Assays; Thermo Fisher) [18,19]. In the final step, the identified genes and proteins will be quantified together with known inflammatory markers (tumor necrosis factor-α, interleukin-6 [IL-6], and interleukin-10) from serum samples using ELISA or the LegendPlex assay (BioLegend) [20]. The blood plasma obtained during cell isolation will also be used for measuring tumor necrosis factor-α and other cytokines (IL-6, interleukin-10, etc) via ELISA. In addition, neuron-specific enolase and S-100 β protein levels will be determined from the remaining blood preoperatively and 48 hours and 5 days postoperatively. The remaining DNA, RNA, and plasma samples will be stored at −20 °C and −80 °C to ensure the possibility of repeat measurements in case of technical errors. The samples will also be preserved for further validation of significant proteins from the proteomic analysis using ELISA and to investigate their genetic basis (polymorphisms and DNA methylation).

### Clinical Data Collection and Processing

A formal Data Monitoring Committee is not required for this study, as there are no interventions being tested and no anticipated risks to participants beyond standard clinical care. Therefore, no interim analyses or stopping guidelines are planned.

The study conduct will be monitored by the study physician and the research team according to standard operating procedures to ensure data quality and protocol adherence. Since there are no safety concerns beyond routine care, no additional independent monitoring is planned.

Clinical parameters are systematically documented and collected throughout the project. These parameters are exported from the routine medical documentation within the various subsystems of the electronic health records. On regular wards, this will be done through the Hospital Information System, and in ICUs, through the Patient Data Management System, capturing the entire course of patient treatment. The clinical data include vital parameters, medications, diagnoses, procedures, and laboratory values. Anesthesia documentation is supplemented by a digital Anesthesia Information Management System, which closely monitors all perioperative anesthesia parameters, including medication, vital signs, and ventilation parameters. Intraoperative electroencephalogram data, recorded during anesthesia depth monitoring, will also be captured.

These routinely collected data will be supplemented by explicitly requested variables to determine study end points, such as delirium scores. For this purpose, a study nurse will assess delirium preoperatively, upon transfer from the recovery room, and on the 5 subsequent postoperative days using the CAM, or CAM-ICU if the patient is transferred to the ICU. A learning effect from repeated testing is only to be expected in patients without delirium. Other outcome parameters include neuropsychological status measured by the Repeatable Battery for the Assessment of Neuropsychological Status, health-related quality of life assessed with the 36-item Short Form Health Survey, and mortality on the day of hospital discharge, as well as on days 30 and 90 after surgery.

These primary clinical and scientific data are then processed by a data scientist using a data integration pipeline to ensure that they are prepared for further analysis. This clinical data will be prepared for further analysis steps as outlined in Integrative Data Analysis and Development of a Delirium Prediction Model. To achieve this, an ETL (extract, transform, load) process will be developed. The aim is to automate this process so it can be utilized in subsequent research projects. A core dataset will be defined, representing comprehensive clinical parameters within a standardized data schema and unified semantics. This will be based on the OMOP CDM (Observational Medical Outcomes Partnership Common Data Model) standard from the Observational Health Data Sciences and Informatics initiative.

The OMOP CDM standard includes the representation of clinical content using standardized vocabularies, enabling the mapping of primary data from various data standards (eg, International Classification of Diseases, 10th Revision [ICD-10] and German Procedure Classification System [OPS]) and facilitating conversion to modern data ontologies such as SNOMED-CT (Systematized Nomenclature of Medicine—Clinical Terminology) and/or LOINC (Logical Observation Identifiers Names and Codes). Furthermore, OMOP CDM supports the eventual integration into the developing research data infrastructure of the Medical Faculty at Ruhr University Bochum, potentially using Health Level Seven Fast Healthcare Interoperability Resources (HL7 FHIR) profiles. This infrastructure constitutes the integration pathway for clinical deployment. Clinical input data (eg, demographics, vital signs, laboratory values, medications, and delirium assessments) are captured automatically from the Hospital Information System, Patient Data Management System, and Anesthesia Information Management System without additional manual data entry by clinical staff, and risk predictions are generated automatically within existing workflows at the point of care. For the preoperative clinical model, all input variables are documented as part of standard preanesthesia assessment, and no laboratory analyses are required, meaning a risk prediction is available immediately at the time of the outpatient consultation with no analytical turnaround time. For the integrative postoperative model, predictions are updated automatically as new data are entered into the electronic record, ensuring timely availability for clinical decision-making throughout the hospital stay.

The processed clinical data will then be combined with data from subsequent work packages to support the development of an artificial intelligence (AI) model. Individual deidentified participant data (including the data dictionary), statistical code, and any other materials will be accessible to authorized personnel of the AI Center.

### Integrative Data Analysis and AI Model Development

This section describes the planned integration and analysis of clinical and multiomics data for the exploratory investigation of potential predictors of POD. Data will be analyzed both independently and in combination with a focus on patient groups with and without POD. Prior to analysis, clinical datasets will be preprocessed; for example, variables with more than 30% missingness will be discarded, whereas remaining gaps will be imputed, where applicable, when missing at random, using multiple imputation by chained equations or other suitable methods. Clinical continuous variables will be centered and scaled to *z* scores, and categorical variables will be one-hot encoded. In proteomics, the analysis will involve deriving accurate quantitative protein data from peptide data, particularly by improving the utilization of shared peptides found in multiple proteins.

Univariate analyses, such as *t* tests and ANOVA (with adjustments for multiple comparisons), will be applied to identify candidate features, which may subsequently be subjected to pathway and enrichment analyses to generate mechanistic hypotheses regarding molecular processes potentially involved in POD. Multivariate techniques, including principal component analysis and clustering, will be applied to uncover latent patient phenotypes beyond a priori groupings.

The significant findings from clinical and omics data are fed into a knowledge base for targeted searches, facilitating focused investigations (targeted proteomics). The entire dataset is also analyzed using multivariate methods, including principal component analysis and clustering, to reveal latent patient phenotypes beyond a priori groupings. Supervised prediction of POD will rely on a benchmark suite of algorithms (eg, regularized logistic regression, random forests, support vector machines, and gradient-boosting methods). Model training and hyperparameter tuning will follow a nested cross-validation scheme, supplemented by a temporally separated holdout set when sufficient data are available. Discrimination and calibration will be reported with area under the receiver operating characteristic curve, area under the precision-recall curve, sensitivity, specificity, accuracy, and *F*-score. The final modeling protocol will be chosen on the basis of performance, interpretability, and clinical plausibility and can be iteratively updated as additional data accumulate.

Given the limited sample size relative to the high-dimensional omics data, all predictive models are exploratory and hypothesis-generating. External validation in independent cohorts will be necessary to confirm predictive performance and clinical utility. Models trained on clinical variables alone will be benchmarked alongside multiomics models for exploratory comparison, reflecting potential applicability in settings where omics data are not routinely available.

The final operating threshold will be selected to prioritize either sensitivity (screening scenario) or specificity (confirmatory setting), as dictated by the clinical use case. Alongside the integrative models, versions limited to routinely available clinical variables will be trained and benchmarked to ensure bedside applicability where omics data are not regularly available. To interpret the resulting models, feature-attribution techniques such as SHAP (Shapley Additive Explanations) values will quantify and visualize each variable’s influence on the prediction, thereby generating mechanistic hypotheses about the molecular and functional pathways leading to POD. To clarify the clinical deployment strategy, the study is designed to yield 2 distinct and complementary model types. The first is a purely preoperative clinical model, trained exclusively on variables available at the preanesthesia outpatient clinic, specifically the DRAS, Mini-Mental State Examination score, age, comorbidities, surgical type, and medication profile. This model requires no blood sampling and can generate a risk prediction at the time of the outpatient consultation, typically days to weeks before surgery. This is the primary bedside instrument of the study and the relevant window for initiating preventive measures, including medication review and reduction of anticholinergic burden, sleep hygiene counseling, optimization of sensory aids, cognitive stimulation, and planning of early postoperative mobilization. The second is an integrative multimodal model incorporating longitudinal biomarker data from all 4 perioperative collection time points. This model serves a distinct purpose: continuous refinement of risk estimates throughout the hospital stay, enabling early detection of evolving POD in the postoperative period. Postoperative biomarker data are therefore not a prerequisite for preoperative risk stratification but an additional layer extending clinical utility into the postoperative setting.

### Comparison With Existing POD Prediction Models

Several prediction models for POD have been developed over the past decade, including the Validation of Prediction of Delirium in ICU Patients (PRE-DELIRIC) model, models based on inflammatory biomarkers, and preliminary ML approaches using single-omics or clinical variables. These models have provided important insights into POD risk factors, but they are generally limited by reliance on either clinical variables alone or a single biomarker modality and often do not account for the complex, multifactorial pathophysiology of delirium.

The CONFUSED study aims to extend beyond these approaches by integrating multiple layers of omics data—proteomics, transcriptomics, and epigenetics—with detailed clinical variables. This integrative, multimodal approach allows for the identification of candidate biomarker signatures and mechanistic pathways associated with POD, which cannot be captured by single-omics or clinical-only models. Advanced ML techniques, including feature selection, regularization, and nested cross-validation, are applied to ensure robustness and interpretability of exploratory predictive patterns.

While this study does not yet provide a systematic performance comparison with existing POD prediction models, it is designed to generate hypotheses and candidate signatures that can inform future external validation studies. By combining diverse molecular and clinical data with interpretable ML, the CONFUSED study seeks to provide incremental value in understanding POD risk and to lay the groundwork for the development of more comprehensive and mechanistically informed predictive models.

### Dissemination Plan

The results of the CONFUSED study will be disseminated through peer-reviewed scientific publications and presentations at national and international conferences in the fields of anesthesiology, perioperative medicine, and neurocognitive research. In addition, the study findings will contribute to ongoing research collaborations and may support the development of future multicenter studies, focusing on biomarker-based prediction and prevention of POD.

## Results

### Overview

The study was registered in the German Clinical Trials Register (DRKS00033854) on March 18, 2024. Patient recruitment commenced in January 2024 and is ongoing at the time of manuscript submission (Table 2). As of now, 135 of the approximately planned 200 to 300 patients have been enrolled, corresponding to approximately two-thirds of the target sample size. Sample collection and laboratory analyses are ongoing. Enrollment is proceeding according to the projected timeline (Table 1), and no major protocol deviations have occurred to date. The first results are expected in July 2026.

**Table 2.** Timeline of the study.

Item	Details
Initial ethical approval	Approved in May 2023
Funding date	Funded in January 2023
Ethical approval amendment	Approved in March 2024
Trial registration	Registered in the German Clinical Trials Register (DRKS00033854) on March 18, 2024
Start of recruitment	Recruitment started in January 2024
End of recruitment	Recruitment is expected to be completed in December 2026
Sample size status	As of February 2026, a total of 135 participants have been enrolled
Data collection period	Data collection is planned from April 2024 to March 2027
Laboratory analyses	Laboratory analyses commenced in February 2026
Statistical analysis	Statistical analysis is scheduled to begin in July 2026
Expected publication	First results are expected to be published in Spring 2027

Biospecimen collection at all 4 predefined perioperative time points is ongoing. Sample processing, including initial proteomic and transcriptomic preparation steps, has been initiated in parallel with recruitment to ensure standardized handling and storage conditions. Comprehensive laboratory analyses are currently underway.

Data cleaning and database validation procedures are being performed continuously. Final data lock is anticipated after the completion of recruitment. Statistical analyses, including integrative multiomics evaluation and the development of predictive models, began in January 2026. First results are expected in March 2026, with dissemination through peer-reviewed publications planned thereafter.

### Trial Status

The first patient was recruited on January 15, 2024. The inclusion of participants is ongoing and is expected to continue until November 2026. The first results are expected in July 2026. The trial protocol and statistical analysis plan can be accessed through the corresponding author.

## Discussion

### Principal Findings

The CONFUSED study is designed to improve the prediction and understanding of POD by integrating multimodal molecular data with detailed clinical information. By combining proteomic, transcriptomic, and epigenetic analyses with perioperative clinical variables, the study aims to identify biomarkers and molecular signatures associated with the development of POD. We anticipate that this integrative approach will enable the identification of biological pathways involved in delirium pathogenesis and support the development of predictive models that may help identify patients at increased risk for POD.

The application of ML techniques to multimodal datasets may further enhance predictive performance compared with traditional statistical models. Ultimately, the findings of the CONFUSED study may contribute to a better understanding of the biological mechanisms underlying POD and support the development of personalized prevention strategies in perioperative medicine.

### Comparison With Prior Work

Previous studies investigating biomarkers of POD have primarily focused on individual biomarkers, such as inflammatory cytokines, neurodegenerative markers, or selected genetic variants. While these approaches have provided valuable insights into potential mechanisms, their predictive performance has often been limited due to the complex and multifactorial nature of delirium.

Recent advances in high-throughput molecular technologies have enabled the investigation of complex biological processes using multiomics approaches. However, studies integrating proteomic, transcriptomic, and epigenetic data in the context of POD remain scarce. The CONFUSED study aims to address this gap by applying a comprehensive multiomics strategy combined with advanced data integration methods. This approach may allow for the identification of novel biomarker panels and biological pathways that cannot be detected using single-omics analyses alone.

The application of AI in medical research has the potential to revolutionize our approach to complex conditions, such as POD. POD, a prevalent and challenging complication following surgery, is characterized by acute cognitive disturbances and fluctuating consciousness [21]. Despite substantial research efforts, the prediction and diagnosis of POD remain difficult due to its multifactorial nature and variable clinical presentation [22,23]. Recent advances in OMICs research combined with AI offer promising solutions to these challenges by enabling more accurate and comprehensive predictive modeling [24].

POD is influenced by a range of factors, including patient age, preexisting cognitive impairment, type of surgery, anesthesia, and inflammatory responses [11]. Traditionally, predicting POD has relied on clinical assessment tools and risk scores, which are often inadequate due to their subjective nature and the heterogeneity of delirium symptoms [4]. Omics data, including genetic, epigenetic, proteomic, and metabolic indicators, offer valuable insights into the biological processes underlying POD. For example, inflammatory proteins such as C-reactive protein and IL-6 or high mobility group box 1 have been linked to the increased risk of delirium [25]. Genetic variants and epigenetic modifications, such as changes in DNA methylation, also contribute to individual susceptibility [26].

### Strengths and Limitations

The CONFUSED study has several strengths. First, it employs a prospective observational design with standardized perioperative data collection and systematic delirium assessments using validated instruments (CAM and CAM-ICU). Second, blood samples are collected at multiple perioperative time points, enabling the investigation of dynamic molecular changes associated with the onset and progression of delirium.

Third, the study integrates multiple layers of molecular data, including proteomics, transcriptomics, DNA methylation, and genetic polymorphisms. This multiomics approach provides a comprehensive view of the biological processes involved in the delirium development. Finally, the application of ML methods for data integration and predictive modeling may improve the identification of clinically relevant biomarker signatures.

Several limitations should be considered. First, the study was conducted at a single center, which may limit the generalizability of the findings to other clinical settings or patient populations. Second, although the planned sample size is sufficient for exploratory analyses, validation in independent cohorts will be necessary to confirm the predictive performance of the identified biomarkers and models.

Third, the high dimensionality of multiomics data combined with a relatively moderate sample size may increase the risk of overfitting in predictive modeling. To address this challenge, appropriate statistical methods, cross-validation strategies, and feature selection approaches will be applied. Finally, while peripheral blood samples provide valuable information about systemic biological processes, they may not fully capture neurobiological mechanisms occurring within the central nervous system.

Other challenges should be considered. Privacy and security concerns, the need for extensive and diverse datasets, and the complexity of translating AI findings into clinical practice are notable hurdles [27-29]. While AI can identify associations and patterns, understanding the underlying mechanisms of POD and establishing causality requires further research.

Future directions should focus on improving the interpretability of AI models, integrating them into clinical workflows, and considering ethical aspects. Collaborative efforts between data scientists, clinicians, and researchers are essential to validate AI-driven predictive models and ensure their successful application in clinical settings. By overcoming these challenges, AI has the potential to significantly improve the accuracy of POD prediction and facilitate personalized interventions, ultimately improving patient outcomes and reducing the burden of POD. With regard to cost-effectiveness, a formal health-economic analysis lies beyond the scope of this protocol study, but several considerations are relevant. First, the preoperative clinical model relies exclusively on routinely collected clinical data and requires no additional laboratory analyses, thereby incurring no additional diagnostic costs. Second, POD is associated with a well-documented economic burden, including prolonged hospitalization, higher rates of institutionalization, extended mechanical ventilation, and long-term cognitive decline. Targeted nonpharmacological prophylaxis initiated following early identification of high-risk patients has been shown in randomized trials to reduce POD incidence and shorten hospital stays, yielding downstream cost savings that are likely to outweigh the costs of screening. Third, the full proteomic and transcriptomic workup performed in this study is explicitly designed to identify a focused panel of high-yield biomarkers, which is anticipated to inform the development of targeted, lower-cost point-of-care assays for future routine clinical implementation, substantially reducing per-patient costs relative to the comprehensive analytical workup performed during the study phase.

### Future Directions

The findings of the CONFUSED study may provide the basis for future research aimed at validating identified biomarkers and predictive models in independent multicenter cohorts. In addition, the identified molecular pathways may represent potential targets for preventive or therapeutic interventions.

In the long term, the integration of molecular biomarkers with clinical risk factors could enable the development of personalized perioperative risk assessment tools and targeted prevention strategies for POD.

## Abbreviations

AI: artificial intelligence
CAM: Confusion Assessment Method
CAM-ICU: Confusion Assessment Method for the intensive care unit
DRAS: Delirium Risk Assessment Score
ELISA: enzyme-linked immunosorbent assay
ETL: extract, transform, load
HL7 FHIR: Health Level Seven Fast Healthcare Interoperability Resources
*ICD-10*: *International Classification of Diseases, 10th Revision*
ICU: intensive care unit
IL-6: interleukin-6
LOINC: Logical Observation Identifiers Names and Codes
ML: machine learning
OMOP CDM: Observational Medical Outcomes Partnership Common Data Model
OPS: German Procedure Classification System
POD: postoperative delirium
PRE-DELIRIC: Validation of Prediction of Delirium in ICU Patients
SHAP: Shapley Additive Explanations
SNOMED CT: Systematized Nomenclature of Medicine—Clinical Terminology
STROBE: Strengthening the Reporting of Observational studies in Epidemiology
TRIPOD: Transparent Reporting of a multivariable prediction model for Individual Prognosis or Diagnosis

## References

[1] MacLullich AMJ, Beaglehole A, Hall RJ, Meagher DJ (2009). Delirium and long-term cognitive impairment. Int Rev Psychiatry.

[2] Scott JE, Mathias JL, Kneebone AC (2015). Incidence of delirium following total joint replacement in older adults: a meta-analysis. Gen Hosp Psychiatry.

[3] Schiemann A, Hadzidiakos D, Spies C (2011). Managing ICU delirium. Curr Opin Crit Care.

[4] Dunne SS, Coffey JC, Konje S (2021). Biomarkers in delirium: a systematic review. J Psychosom Res.

[5] Maldonado JR (2013). Neuropathogenesis of delirium: review of current etiologic theories and common pathways. Am J Geriatr Psychiatry.

[6] Schaefer ST, Koenigsperger S, Olotu C, Saller T (2019). Biomarkers and postoperative cognitive function: could it be that easy?. Curr Opin Anaesthesiol.

[7] Wang Y, Chen Z, Zhao Y (2013). Epigenetics as a new therapeutic target for postoperative cognitive dysfunction. Med Hypotheses.

[8] Joris J, Kehlet H, Slim K (2022). Postoperative cognitive dysfunction: time for enhanced recovery after surgery programmes. Eur J Anaesthesiol.

[9] Vutskits L, Xie Z (2016). Lasting impact of general anaesthesia on the brain: mechanisms and relevance. Nat Rev Neurosci.

[10] Hudson AE, Hemmings HC (2011). Are anaesthetics toxic to the brain?. Br J Anaesth.

[11] Liu X, Yu Y, Zhu S (2018). Inflammatory markers in postoperative delirium (POD) and cognitive dysfunction (POCD): a meta-analysis of observational studies. PLoS One.

[12] Inouye SK, Westendorp RGJ, Saczynski JS (2014). Delirium in elderly people. Lancet.

[13] Riker RR, Fraser GL (2020). Delirium-beyond the CAM-ICU. Crit Care Med.

[14] Ho MH, Nealon J, Igwe E (2021). Postoperative delirium in older patients: a systematic review of assessment and incidence of postoperative delirium. Worldviews Evid Based Nurs.

[15] Gusmao-Flores D, Salluh JIF, Chalhub RÁ, Quarantini LC (2012). The confusion assessment method for the intensive care unit (CAM-ICU) and Intensive Care Delirium Screening Checklist (ICDSC) for the diagnosis of delirium: a systematic review and meta-analysis of clinical studies. Crit Care.

[16] Chen J, Liang S, Wei M (2024). Trace of delirium after robotic lower abdominal tumor resection at different end-tidal carbon dioxide: a RCT trial. BMC Anesthesiol.

[17] Rump K, Unterberg M, Dahlke A (2019). DNA methylation of a NF-κB binding site in the aquaporin 5 promoter impacts on mortality in sepsis. Sci Rep.

[18] Rahmel T, Nowak H, Rump K (2019). The aquaporin 5 -1364A/C promoter polymorphism is associated with cytomegalovirus infection risk in kidney transplant recipients. Front Immunol.

[19] Rahmel T, Nowak H, Rump K, Siffert W, Peters J, Adamzik M (2018). The aquaporin 5 -1364A/C promoter polymorphism impacts on resolution of acute kidney injury in pneumonia evoked ARDS. PLoS One.

[20] Ziehe D, Marko B, Thon P (2024). The aquaporin 3 polymorphism (rs17553719) is associated with sepsis survival and correlated with IL-33 secretion. Int J Mol Sci.

[21] Cascella M, Muzio MR, Bimonte S, Cuomo A, Jakobsson JG (2018). Postoperative delirium and postoperative cognitive dysfunction: updates in pathophysiology, potential translational approaches to clinical practice and further research perspectives. Minerva Anestesiol.

[22] Holtkamp C, Koos B, Unterberg M (2019). A novel understanding of postoperative complications: in vitro study of the impact of propofol on epigenetic modifications in cholinergic genes. PLoS One.

[23] Rump K, Adamzik M (2022). Epigenetic mechanisms of postoperative cognitive impairment induced by anesthesia and neuroinflammation. Cells.

[24] Han T, Xiong F, Sun B, Zhong L, Han Z, Lei M (2024). Development and validation of an artificial intelligence mobile application for predicting 30-day mortality in critically ill patients with orthopaedic trauma. Int J Med Inform.

[25] Zou Y, Wu Y, Wei A (2025). Serum HMGB1 as a predictor for postoperative delirium in elderly patients undergoing total hip arthroplasty surgery. Adv Clin Exp Med.

[26] Nishizawa Y, Yamanashi T, Nishiguchi T (2024). The genome-wide DNA methylation changes in gastrointestinal surgery patients with and without postoperative delirium: evidence of immune process in its pathophysiology. J Psychiatr Res.

[27] Cho H, Froelicher D, Dokmai N (2024). Privacy-enhancing technologies in biomedical data science. Annu Rev Biomed Data Sci.

[28] El Azzouzi M, Bellafqira R, Coatrieux G, Cuggia M, Bouzille G (2024). Secure extraction of personal information from EHR by federated machine learning. Stud Health Technol Inform.

[29] Xu T, Weng H, Liu F (2024). Current status of ChatGPT use in medical education: potentials, challenges, and strategies. J Med Internet Res.

